# Retrograde cerebral embolism and pulmonary embolism caused by patent ductus arteriosus: a case report

**DOI:** 10.1186/s13019-024-02901-w

**Published:** 2024-06-27

**Authors:** Jian Li, Tihua Liu, Qinfeng Rong

**Affiliations:** Department of Neurology, The People’s Hospital of Huantai County (The 9 th People’s Hospital of Zibo), No. 2198 Huantai Avenue, Suo Town, Huantai Country, Zibo, Shandong Province 256400 China

**Keywords:** Acute ischemic stroke, Patent ductus arteriosus, Paradoxical embolism, Pulmonary embolism, Catheter-directed thrombolysis, Case report

## Abstract

**Background:**

Although rare, paradoxical embolism sometimes occurs with patent ductus arteriosus (PDA). This study presents a case of PDA-associated paradoxical embolism with acute ischemic stroke (AIS) and pulmonary embolism (PE) following thoracoscopic surgery.

**Case Presentation:**

A 65-year-old woman developed acute-onset aphasia and right hemiparesis on the third day following thoracoscopic resection for a right lung tumor. Brain magnetic resonance imaging revealed multiple infarcts, and lower extremity venous Doppler ultrasound revealed deep vein thrombosis. The patient subsequently developed dyspnea, tachycardia, and hypoxemia. PE was confirmed by percutaneous transfemoral venous selective pulmonary angiography, which meanwhile demonstrated a PDA lesion. The patient, after receiving catheter-directed thrombolysis and inferior vena cava filter placement, improved in both neurological and respiratory status.

**Conclusion:**

For an uncommon but potentially fatal case with PDA-induced paradoxical embolism causing AIS and PE, early recognition and treatment are vital. Further studies are warranted to determine the optimal management and prognosis of patients with PDA-related embolic events.

**Supplementary Information:**

The online version contains supplementary material available at 10.1186/s13019-024-02901-w.

## Background

Acute ischemic stroke (AIS) is a major global challenge. In China, the stroke mortality rate is four times higher than that in Europe and the United States [1], highlighting the need for improved understanding and management of this condition in different populations and settings.

Paradoxical embolism represents a relatively rare but devastating AIS subtype, characterized by a complex thromboembolic trajectory that involves both systemic and pulmonary circulations [2]. The prevalence of paradoxical embolism in ischemic stroke, according to documented postmortems, varies from 7 to 40% [3]. Usually, paradoxical embolism arises in patients with venous thromboembolism, which originates in lower extremities and might lead to neuroarterial embolization [4]. Among the possible intracardiac sources, patent foramen ovale (PFO) has been extensively reported and studied while patent ductus arteriosus (PDA) is seldom referred to [5].

The concurrence of acute pulmonary embolism (PE) is a strong predictor of poor prognosis and high in-hospital mortality for patients with AIS [6], emphasizing the importance of timely diagnosis and treatment. Unfortunately, due to variable and nonspecific presentations, nearly half the PE cases associated with progressive stroke remain undiagnosed until death [7].

Here, we present a unique case of PDA with retrograde cerebral embolization and PE following thoracic surgery. Based on a rapid and accurate diagnosis, the patient underwent neurointerventional recanalization and anticoagulation therapy, attaining a favorable outcome.

## Case presentation

**Fig. 1:**
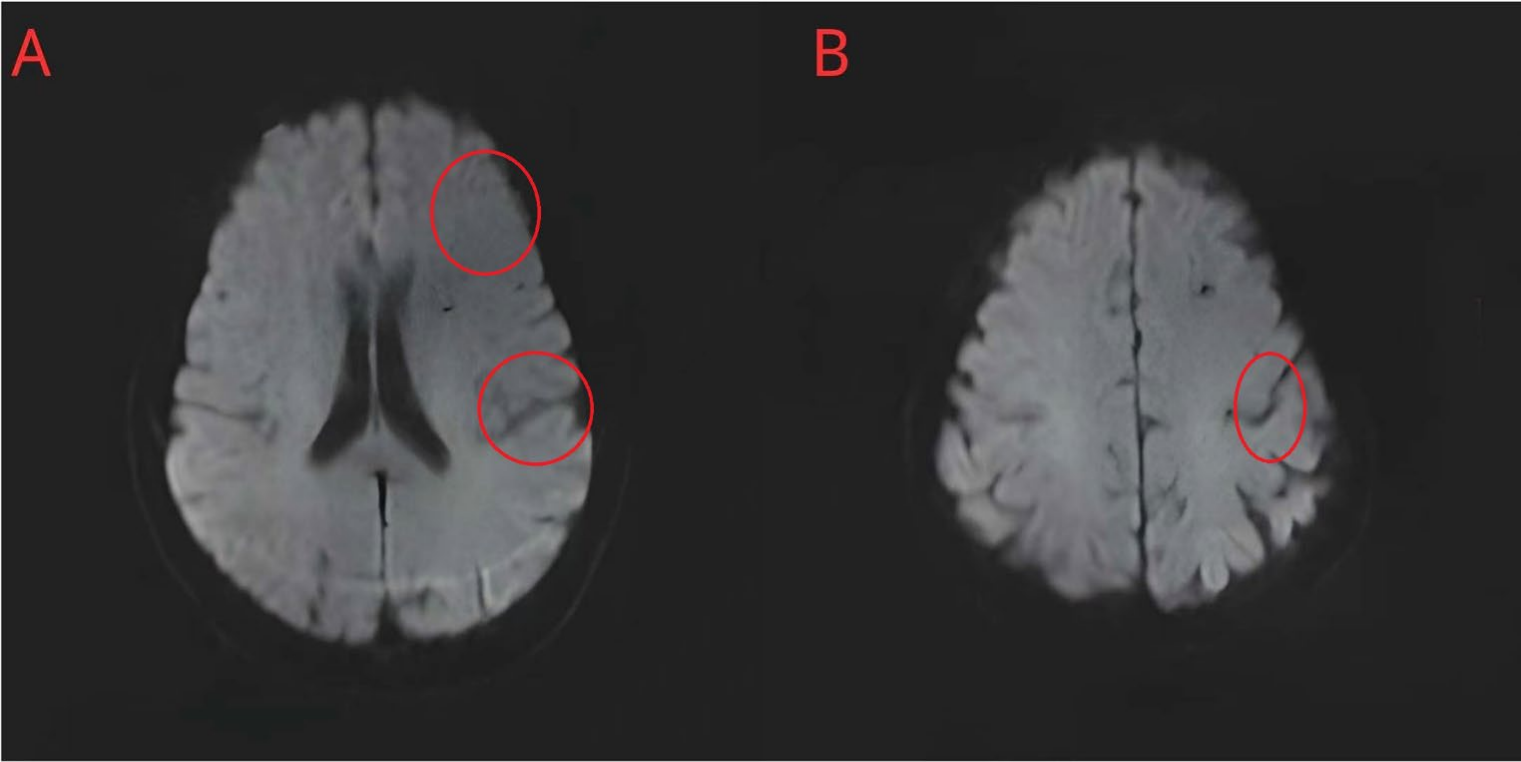
Preoperative brain MRI. The MRI shows low signal intensity in both cerebral hemispheres on DWI (red circles) without significant infarct lesions in the left frontal lobe (**A**) or at the junction of the left frontoparietal lobes (**B**). This indicates that the patient had no cerebrovascular abnormalities before thoracic surgery

A 65-year-old female patient was admitted to the thoracic surgery department on November 29, 2021 for a right lung nodule. She denied any significant past medical history and was generally healthy. A detailed enhanced chest computed tomography (CT) scan validated the existence of the nodule (a suspected malignant tumor) in the upper lobe of her right lung. A surgical resection was arranged for treatment. Preoperative brain magnetic resonance imaging (MRI) showed multiple low-signal lesions in the diffusion-weighted imaging (DWI) of both cerebral hemispheres of the brain (Fig. 1), while lower extremity venous Doppler ultrasound results were unremarkable.

On December 4, 2021, she underwent a successful thoracoscopic resection (under general anesthesia) of the apical posterior segment of the right upper lobe and middle lobe nodule, along with lymph node dissection. Postoperative monitoring and symptomatic treatment, including oxygen therapy, were normal.

Unexpectedly, at 16:10 (on December 7, 2021), the patient developed sudden speech difficulty and right-sided weakness, and was promptly diagnosed with AIS. Physical examination revealed coarse breath sounds in both lungs (accentuated on the right side) and moist rales. Her vital signs remained stable, with a heart rate of 75 beats per minute (bpm) and blood pressure at 117/72 mmHg. Cardiac, vascular, and abdomen examinations were unremarkable. No edema was present in the lower extremities.

However, neurological examination identified defective consciousness and speech. Cranial nerve functions remained largely intact, except for decreased pain and temperature sensations over the right face. Tongue deviation to the right was also noted. There was a significant difference in muscle strength between sides: grade 1 weakness on the right compared to normal grade 5 strength on the left. Her muscle tone was normal without involuntary movements. The skin examination was unremarkable. Coordination testing revealed impairment on her right side. Reflexes were symmetric overall, except for the presence of a positive Babinski sign and a positive Chaddock sign on the right side. The National Institute of Health Stroke Scale (NIHSS) score was 14 points (level of consciousness: 1 point; language: 1 point; facial palsy: 2 points; right arm: 4 points; right leg: 4 points; ataxia: 1 point; sensory: 1 point). Meanwhile, the Modified Rankin Scale (mRS) score of 4 points underscored severe disability. However, the Modified Framingham Stroke Scale (MFSS) scored only 2 points. Additionally, the Water swallow test showed grade I.

**Fig. 2:**
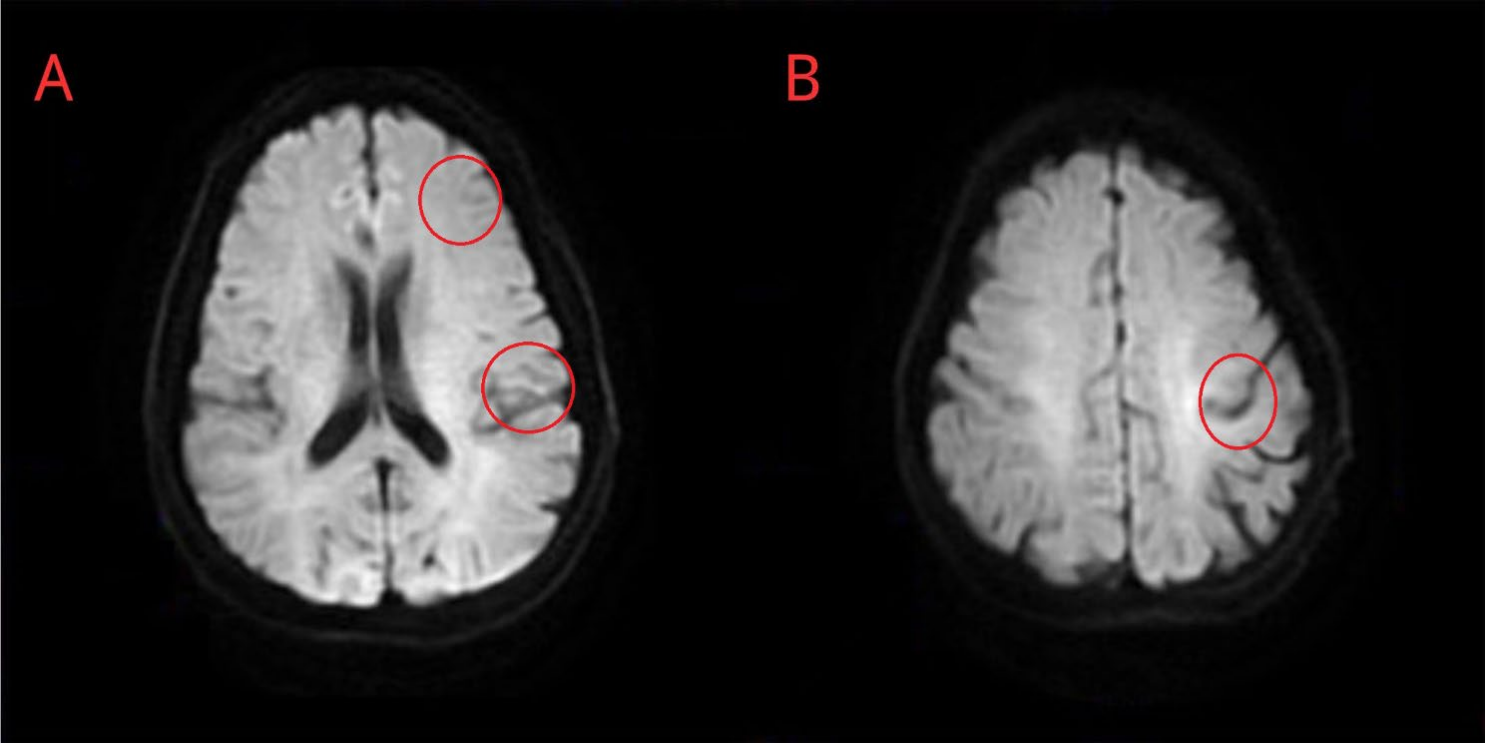
Brain MRI after AIS. The MRI demonstrates patchy signal intensity (red circles) in the left frontal lobe (**A**) and at the junction of the left frontoparietal lobes (**B**) on DWI, suggesting the presence of cerebral infarction at a very early stage

Subsequent brain MRI unveiled multiple patchy lesions in DWI across both cerebral hemispheres (Fig. 2), which conform to the image features in stroke of other determined etiology (SOE) at an early stage.

**Fig. 3:**
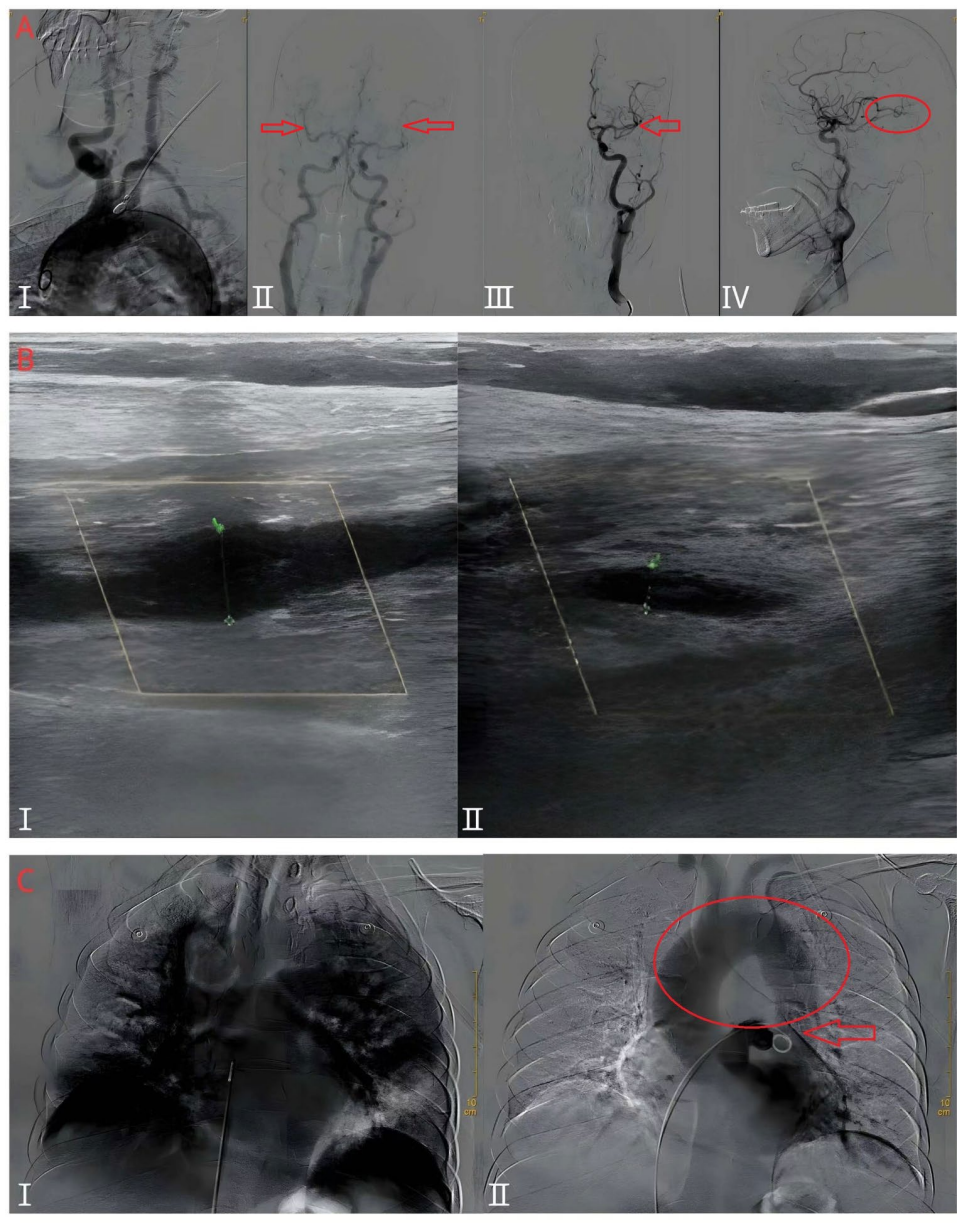
Vascular imaging during AIS and PE. (**A**) Cerebral angiograms showing (**I**) extracranial contrast of anterior arch (type I) with no significant stenosis of the bilateral subclavian, internal carotid, and vertebral arteries (red arrows); (**II**) intracranial contrast angiography depicting no stenosis of the right middle cerebral artery but unclear distal filling of the left middle cerebral artery M2 superior trunk (red arrows); (**III**) anterior view of a left carotid artery demonstrating unclear distal filling of the left middle cerebral artery M2 superior trunk (red circles); and (**IV**) lateral view of left carotid artery indicating unclear distal filling of the left middle cerebral artery M2 superior trunk, suggesting possible thrombosis. (**B**) Lower extremity venous Doppler ultrasounds revealing (**I**) expanded muscular veins behind the right calf (maximum diameter ~ 9.2 mm) with hypoechoic nonvascular cavities, and (**II**) similar expanded hypoechoic venous cavity behind the left calf (maximum diameter ~ 4.0 mm), indicating deep vein thrombosis. (**C**) Pulmonary arteriography showing partial pulmonary artery embolism (**I**) and PDA (**II**, red circle and red arrow)

The patient presented with persistent stroke symptoms 1 h after onset. Intravenous thrombolysis was not an option due to recent major surgery and ongoing mild bloody chest tube drainage. Cerebral angiography performed at 17:20 showed complete obstruction of the left middle cerebral artery M2 superior division distal branch, indicative of an acute embolism (Fig. 3A). Unfortunately, mechanical thrombectomy was ruled out, as no accessible target was identified. Intra-arterial tirofiban was administered as a treatment for suspected small distal vessel occlusion, while repeat angiography after 15 min showed no improvement.

After the surgery (at 18:15), the patient regained consciousness with notable symptoms, including incomplete motor aphasia, right-sided facial and limb weakness, and a positive right Babinski sign. The NIHSS score was recorded at 8 (language: 1 point; facial palsy: 2 points; right arm: 2 points; right leg: 2 points; and ataxia: 1 point), indicating moderate severity of stroke. Immediate medical interventions included the administration of tirofiban (5 mL/h) for antiplatelet effects, atorvastatin for plaque stabilization, edaravone for neuroprotection, and dibenzyline to enhance collateral circulation. Supportive care and fluid replacement were also promptly initiated to optimize her recovery.

Nevertheless, the patient developed sudden agitation at 21:00, with abnormal vital signs, including tachycardia (heart rate of 120–130 bpm), tachypnea (respiratory rate 30–35 breaths per minute), hypoxemia (blood oxygen saturation as low as 80%), and hypertension (blood pressure of 149–159/95–110 mmHg).

Venous Doppler ultrasound of the bilateral lower extremities revealed deep vein thrombosis **(**Fig. 3B**)**. Percutaneous transfemoral venous selective pulmonary angiography confirmed acute PE and incidentally discovered PDA **(**Fig. 3C**)**. The patient underwent inferior vena cava filter placement and systemic anticoagulation (heparin 2000 IU intravenously) under local anesthesia, which resulted in symptom improvement. Then, the patient received mask oxygenation, immobilization of the bilateral lower extremities, low molecular weight heparin (4250 IU intramuscularly every 12 h), and continuous infusion of tirofiban (5 mL/h) followed by anticoagulation with aspirin. Long-term treatment with low molecular weight heparin and aspirin was given for 2 weeks.

Two weeks after initiating anticoagulation and antiplatelet therapies, the patient showed significant improvement in neurological and functional status. At the follow-up examination, she was alert and oriented, with near-complete resolution of prior dysarthria. Her muscle strength improved to grade 4 + on the right and normal grade 5 on the left. Sensation and coordination were intact, except for a lingering positive Babinski sign on the right. Serum biomarkers, including cardiac troponin and D-dimer, were also normalized. Her NIHSS score was reduced to 3 (language: 1 point, right arm: 1 point, right leg: 1 point).

**Fig. 4:**
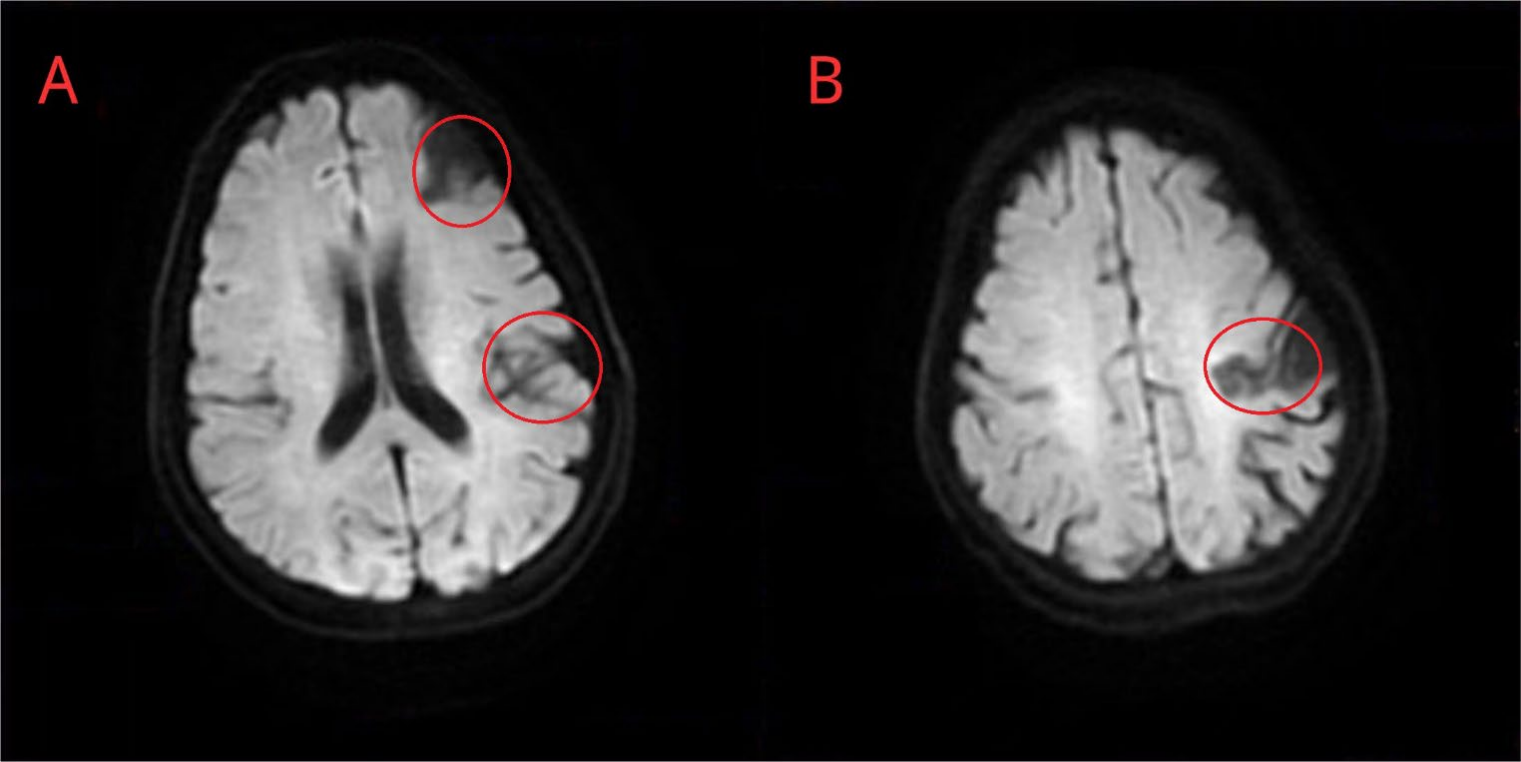
Brain MRI a month after AIS. The MRI reveals brain substance and local fluid signal alterations (red circles) in the left frontal lobe (**A**) and frontoparietal junction (**B**), suggesting liquefactive necrosis of the left frontal and frontoparietal infarcts

The patient was subsequently discharged in stable condition on December 22, 2021 following a regimen of aspirin, anticoagulant, and statin therapy. the electrocardiogram before discharge was normal (Supplemental Fig. S2). One month later, a follow-up brain MRI revealed encephalomalacia and localized cystic signals in the left frontal and frontoparietal junctions (Fig. 4). Cerebral angiography confirmed a left frontal lobe and frontoparietal watershed ischemic stroke due to acute occlusion at the distal end of the left middle cerebral artery M2 segment.

## Discussion and conclusions

We reported a case of paradoxical embolism triggered by PDA, along with AIS and PE, after thoracic surgery. This condition, characterized by a complex thromboembolic pathogenesis involving both systemic and pulmonary circulation, has rarely been reported in the literature [5, 6]. A mortality rate exceeding 60% is attributed to this complex condition [6]. In our case, the patient met the diagnostic criteria for paradoxical embolism, characterized by venous source thromboembolism and right-to-left cardiac shunting due to a structural anomaly. Additionally, unique clinical features were observed. The complex pathophysiological mechanisms of paradoxical embolism resulting from PDA are highlighted by the detailed interplay between systemic and pulmonary circulation during thromboembolic events [8].

First, we noted a significant improvement in symptoms and partial recanalization following the injection of tirofiban into the distal internal carotid artery during angiography [9]. This observation is pivotal, as it hints at the potential efficacy of high-pressure delivery of contrast agents in disrupting distal cerebral emboli, typically resistant to chemical thrombolysis or mechanical thrombectomy [10]. The rarely studied phenomenon aligns with previous findings [11–13] underscoring enhanced clot dissolution and improved patient outcomes via targeted thrombolytic therapy. The integration of localized drug delivery and mechanical interventions could potentially redefine treatment protocols for distal cerebral emboli and warrant comprehensive investigation to validate their efficacy and safety. Based on the patient’s medical history and clinical manifestation, the factors behind the series of embolisms involved a hypercoagulable state of blood after lung surgery, PDA, and relative high pressure of the pulmonary artery.

**Fig. 5:**
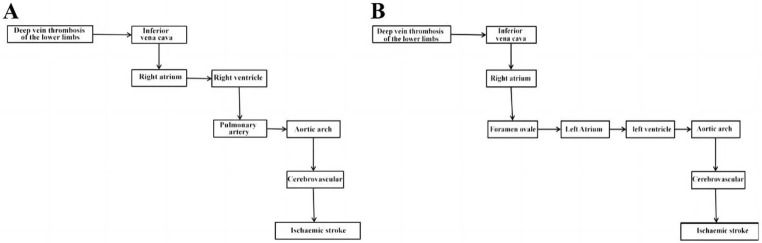
Paradoxical embolism pathway diagram. (**A**) The diagram illustrates the flow direction of the thrombus from PDA to paradoxical embolism. (**B**) The diagram depicts the flow direction of thrombus from PFO to paradoxical embolism

Second, the incidental discovery of PDA on pulmonary angiography is also noteworthy. While PFO is known to be the most common cause **(**Fig. 5B**)**, right-to-left shunting via PDA can occur more readily without the need for pulmonary hypertension or elevated right atrial pressure **(**Fig. 5A**)** [5, 14]. Even if there was a possibility that the tiny emboli moved from pulmonary veins to cerebral arteries along the antegrade flow, the paradoxical embolism through PDA seems more logical, as the cerebral infarction occurred before the pulmonary embolism. Our case highlights the importance of considering this situation, especially in patients with a history of thoracic surgery. Early recognition combined with neuroendovascular intervention might contribute to favorable outcomes, despite the known dismal prognosis.

Furthermore, multimodal imaging plays a vital role in the diagnosis and management of paradoxical embolism caused by PDA. In our case, percutaneous transfemoral venous selective pulmonary angiography was conducted upon suspected pulmonary embolism (PE). In doing so, the aortic arch was meanwhile visualized quickly and clearly, indicating the presence of patent ductus arteriosus (PDA), as well as relatively high pressure of the pulmonary artery. This underlays speculation on the paradoxical embolism. We also performed a transesophageal echocardiogram 1 month after discharge (Supplemental Fig. S1), which evinced an intact atrial septum, effectively ruling out the PFO. These imaging techniques also enabled us to evaluate the severity of the condition, the degree of vascular occlusion, and the effect of the treatment. Additionally, we performed a follow-up brain MRI at 9 months to assess the long-term outcome and the sequelae of the ischemic stroke.

The significance of multimodal imaging in the early diagnosis of conditions such as stroke, PE, and PDA has been confirmed by numerous studies [15–17]. These studies underscore the essentiality of cutting-edge imaging techniques for identifying and facilitating timely interventions. In light of this evidence, we advocate the integration of a multimodal imaging strategy to ensure accurate diagnosis and effective management of these intricate and uncommon conditions.

This report is based on a single observational case study, which limits the ability to establish direct causation due to the absence of a comparison group and randomization. The long-term prognosis remains uncertain and requires further follow-up. Moreover, additional functional and imaging studies could have better characterized patient recovery beyond hospital discharge.

This case report illuminates the complex clinical and academic landscape surrounding the diagnosis and treatment of paradoxical embolism caused by PDA, a rare and devastating disease. The presented case underscores the critical role of prompt disease recognition through multimodal imaging, which enables timely neuro-interventional and anticoagulation therapies that are pivotal in reducing mortality rates.

### Electronic supplementary material

Below is the link to the electronic supplementary material.

Supplementary Material 1

## Data Availability

No datasets were generated or analysed during the current study.

## Abbreviations

AIS: Acute ischemic stroke
bpm: Beats per minute
CT: Computed tomography
DWI: Diffusion-weighted imaging
MFSS: Modified Framingham Stroke Scale
MRI: Magnetic resonance imaging
mRS: Modified Rankin Scale
NIHSS: National Institute of Health Stroke Scale
PDA: Patent ductus arteriosus
PE: Pulmonary embolism
PFO: Patent foramen ovale
SOE: Stroke of other determined etiology
