# Supplementary material for: Retrograde cerebral embolism and pulmonary embolism caused by patent ductus arteriosus: a case report
Source: J Cardiothorac Surg. 2024 Jun 27;19:393. doi: 10.1186/s13019-024-02901-w (PMC11210163; doi:10.1186/s13019-024-02901-w)

Supplemental Fig. 1: Transesophageal echocardiography evinced an intact atrial septum（red circles) to exclude patent ductus arteriosus (PDA).


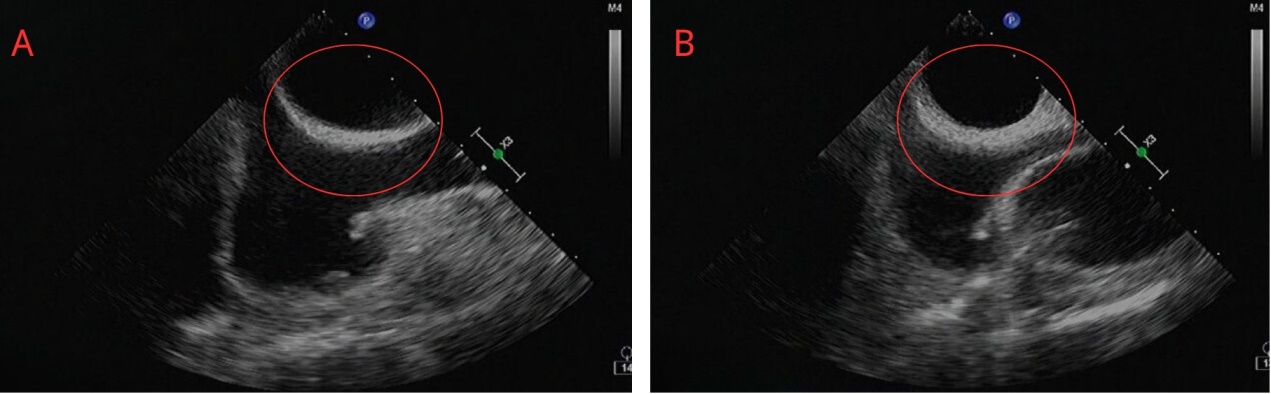


Supplemental Fig. 2: Electrocardiogram of the patient on December 21, 2021 displaying no sign of atrial fibrillation.


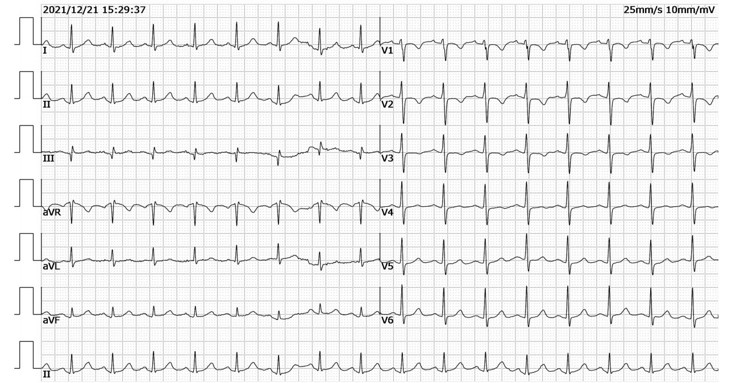

Supplement: Supplementary file 1 — Supplementary Material 1 [file 13019_2024_2901_MOESM1_ESM.docx]
